# Particle Disposition in the Realistic Airway Tree Models of Subjects with Tracheal Bronchus and COPD

**DOI:** 10.1155/2018/7428609

**Published:** 2018-08-05

**Authors:** Baihua Zhang, Shouliang Qi, Yong Yue, Jing Shen, Chen Li, Wei Qian, Jianlin Wu

**Affiliations:** ^1^Sino-Dutch Biomedical and Information Engineering School, Northeastern University, Shenyang, China; ^2^Key Laboratory of Medical Image Computing of Northeastern University (Ministry of Education), Shenyang, China; ^3^Department of Radiology, ShengJing Hospital of China Medical University, Shenyang, China; ^4^Department of Radiology, Affiliated Zhongshan Hospital of Dalian University, Dalian, China; ^5^College of Engineering, University of Texas at El Paso, El Paso, USA

## Abstract

Dispositions of inhalable particles in the human respiratory tract trigger and exacerbate airway inflammatory diseases. However, the particle deposition (PD) in airway of subjects with tracheal bronchus (TB) and chronic obstructive pulmonary diseases (COPD) is unknown. We therefore propose to clarify the disrupted PD associated with TB and COPD using the computational fluid dynamics (CFD) simulation. Totally nine airway tree models are included. Six are extracted from CT images of different individuals (two with TB, two with COPD, and two healthy controls (HC)). The others are the artificially modified models (AMMs) generated by the virtual lesion. Specifically, they are constructed through artificially adding a tracheal bronchus or a stenosis on one HC model. The deposition efficiency (DE) and deposition fraction (DF) in these models are obtained by the Euler-Lagrange approach, analyzed, and compared across models, locations, and particle sizes (0.1-10.0 micrometers). It is found that the PD in models with TB and COPD has been disrupted by the geometrical changes and followed airflow alternations. DE of the tracheal bronchus is higher for TB models. For COPD, the stenosis location determines the effects on DE and DF. Higher DF at the trachea is observed in TB1, TB2, and COPD2 models. DE increases with the particle size, and DE of the terminal bronchi is higher than that of central regions. Combined with AMMs, the CFD simulation using realistic airway models demonstrates disruptions of DP. The methods and findings might help understand the etiology of pulmonary diseases and improve the efficacy of inhaled medicines.

## 1. Introduction

Epidemiologic studies have provided with evidences that exposure to particulate air pollution is associated with the morbidity and mortality of lung cancer, obstructive pulmonary diseases, and cardiovascular diseases [1–3]. Inhalable particles deposit in the human respiratory tract and trigger and exacerbate airway inflammatory diseases [4]. Therefore, understanding the particle deposition (PD) is important because it potentially helps discover the pathophysiological mechanism for the above diseases. Moreover, the aerosol drug therapy is a promising method of diverging drugs [5, 6]. The study of the PD in the lung might help optimize the drug delivery devices and improve the efficacy of inhalation therapy.

The computational fluid dynamics (CFD) simulation is a valuable way to characterize the fluid flow and PD in human airway [7, 8]. CFD simulation can characterize the PD while reducing the cost and time of experiments [9]. The results of CFD have been validated by both in vitro experiments [10–12] and in vivo SPECT/CT images [13].

Some CFD studies on PD have adopted the idealized airway models for they can estimate global deposition [7]. The widely used model is Weibel model. According to principle of this model, each lung generation branches symmetrically into two identical daughter branches [14]. PD in the normal and partially obstructed airway models has been investigated [15–17]. Farkhadnia et al. [18] simulated the PD in airway models with completely blocked bronchi. Some methodological improvements and model details have also been investigated [19, 20].

More investigations have used realistic models after realizing that the accurate and realistic airway models are the necessary precursors for PD analysis [21–25]. These realistic models extracted from CT images are patient-specific and able to represent the airway configurations accurately. Luo and Liu [21] found that the laryngeal and cartilages make the trachea capture a big portion of particles. Regional deposition is dependent on airway geometry, airflow rates, and particle diameter [26]. Alzahrany et al. [27] found that the orientation of endotracheal tube determines the local and total deposition. Katz et al. [28] considered the influence of lung volume during imaging on PD.

Few studies have been conducted on the PD in airway models with diseases. Vinchurkar et al. [29] found that the lower lung deposition occurs for asthmatic patients with higher extrathoracic resistance. Bos et al. [30] studied the antibiotic PD in airways of patients with cystic fibrosis. It is revealed that lower lobes own high PD fraction, more diseased lobes get less drug, and the deposition is highly dependent on the patient-related factors.

The tracheal bronchus (TB) is a rare congenital anomaly characterized by the presence of an abnormal bronchus originating from the trachea or main bronchi and directed toward the upper lobe [31]. Our previous study has shown that high airflow velocity, wall pressure, and wall shear stress present locally at the tracheal bronchus [32]. However, the effect of additional TB on the PD is unknown. Obstructive lung diseases (COPD) are characterized by the obstruction and obliteration of small airway and emphysema [33]. One risk factor of COPD is the exposure of particulate material, resulting in the inflammation of the air tracts [1]. No study on the PD in realistic airway model of subjects with COPD is done, though some works in idealized COPD airway model have been reported [15, 16].

This is the first work of studying the PD in the realistic airway tree models of subjects with TB and COPD. The aim is to clarify the alternation of the PD associated with TB and COPD. Besides six models extracted from CT images, we have also developed other three artificially modified models (AMMs) generated by the virtual lesion. The spatial distributions of the PD in these models are obtained through CFD simulation, analyzed, and compared across models, locations, and particle sizes.

## 2. Materials and Methods

### 2.1. CT Images Acquisition and Model Construction

The data of two TB patients, two COPD patients, and two healthy controls (HC) are from ShengJing Hospital of China Medical University. All participants had given their informed consent for this study and the approval from the hospital review boards had been obtained. For all the acquisitions of CT images, the tube voltage is set as 120 kV, the slice thickness as 1.0 mm, and the reconstruction matrix as 512 × 512. Details on the subjects and CT acquisition parameters are listed in Table 1.

**Figure 1:**
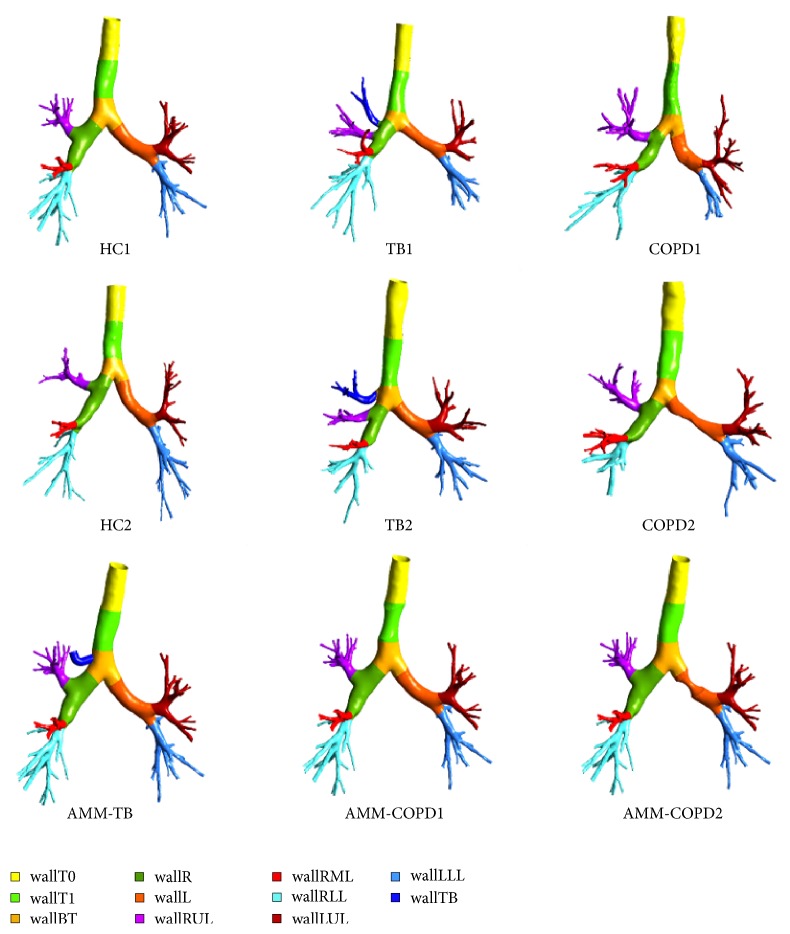
The structures of 9 airway tree models (HC1 and HC2 are extracted from the CT images of two healthy controls, TB1 and TB2 are from two subjects with the tracheal bronchus, and COPD1 and COPD2 are from two subjects with COPD, and AMM-TB, AMM-COPD1, and AMM-COPD2 are the artificially modified models generated by the virtual lesion from HC1).

The airway tree is segmented from the CT images using the algorithm of deep segmentation embedded in the medical imaging process software named Mimics (Materialise Corp, Belgium). The segmentation result is exported as a constructed model in the STL (Standard Tessellation Language) format, and then the model is imported into Geomagic Studio to reduce the number of stripes and smooth the surface. The model is converted into the Parasolid format by SolidWorks (SOLIDWORKS Corp, Waltham, USA). The procedures are the same as those used in our previous studies [32, 34]. Finally, the model is input into ANSYS Workbench 16 to simulate the airflow and PD. The test software is the FLUENT in ANSYS workbench 16.0, and the hardware environment consists of a HP Z820 workstation with two Intel Xeon 2.60GHz CPUs and one 64G RAM. The typical run time is about 2 hours for each case of each subject. The six realistic airway models are given in Figure 1. Some morphometric parameters of the lung airways, such as volume, surface area, and inlet area, are provided in Table 2.

One method named the virtual lesion is introduced to generate three AMMs, with the aim of eliminating interference of the variables resulting from personalization in the airway geometry. Specifically, they are constructed through artificially adding a tracheal bronchus, a stenosis at trachea, and a stenosis at the left main bronchus to the HC1model, as shown in Figure 1. The three models (AMM-TB, AMM-COPD1, and AMM-COPD2) are to simulate the structural modification of TB, COPD1, and COPD2. The stenosis in AMM-COPD1 and AMM-COPD2 is formed by the Boolean operations of a bracelet-shaped volume and the airway model of HC1. The degree of stenosis is 54.7% and 17.8%, respectively.

To quantify the particle deposition, each of the six models (HC1, HC2, COPD1, COPD2, AMM-COPD1, and AMM-COPD2) is further divided into nine regions. Those regions are wallT0, wallT1, wallBT, wallL, wallR, wallRUL, wallRML, wallRLL, wallLUL, and wallLLL. For the remaining three models (TB1, TB2, and AMM-TB), one additional region located on trachea bronchus is named wallTB.

### 2.2. Boundary Conditions and the Flow Model

Since the tidal volume ranges from 350 to 600 ml, in our study, we adopted 500 ml for tidal volume. The total time of respiratory cycle is 5.1 s and the value of inspiration time/expiration time ratio is 1:2. The sine curve of respiratory is a simplified method of representing nature respiratory cycle. The same sinusoidal shaped breathing profile has been adopted by Bos et al. [30] and Qi et al. [34]. We used the maximum flow rate in respiratory cycle as the steady inlet flow rate, and the steady inlet flow rate is calculated to be 26.7 L/minute. This flow rate is the same in each of the nine models. The inlet velocity is calculated according to the inlet flow rate and the inlet area, it ranges from 1.45 to 2.48 m/s. The constant outlet pressure of the atmospheric pressure is set as the outlet boundary condition [35]. No-slip boundary condition at the wall is adopted. In our study, the properties of the inhaled air are set as the default value of air in FLUENT material database, corresponding to the condition of the standard atmosphere and 15°C. Specifically, the air is assumed to be a Newtonian fluid with a constant density of 1.225 kg/m^3^ and a viscosity of 1.7984*∗*10^-5 ^kg/m-s [18].

FLUENT 16.0 is used for solving the governing equations for the airflow and the particle trajectory analysis. The LRN k–*ω* turbulence model is used, and turbulent intensity is chosen as 5% [8]. The LRN k–*ω* model can accurately predict the pressure drops and the velocity profiles [15, 36] and has been proved to be more accurate [10]. A residual with a value less than 10^−6^ is taken as the convergence criterion. The SIMPLEC algorithm is used for the pressure-velocity coupling.

### 2.3. The Discrete Phase Model and Particles Setting

The discrete phase model is used in the analysis of the PD. The particle trajectory is computed through the equation of the balance of forces acting on that particle [22]. The Euler-Lagrangian method is used for unsteady tracking of the particles, as done by Takano et al. [37] and Ilie et al. [38]. The equation describing the particle velocity for a Cartesian coordinate system in the Lagrange formulation has been defined as

(1) $$\begin{array}{c} \frac{\partial u_{p}}{\partial t}=F_{D}\left(\;u-u_{p}\;\right)+\frac{g_{x}\left(\rho_{p}-\rho \right)}{\rho_{p}}+F_{x} \end{array}$$

where *F*_*x*_ is an additional acceleration (force/unit particle mass) term, *F*_*D*_(*u* − *u*_*p*_) is the drag force per unit particle mass, and

(2) $$\begin{array}{c} F_{D}=\frac{18\mu C_{D}{Re}_{r}}{24\rho_{p}d_{p}^{2}} \end{array}$$

In this formula,* u* is the fluid velocity, *u*_*p*_ is the particle velocity,* μ* is the dynamic viscosity of the fluid,* ρ* is the fluid density, *ρ*_*p*_ is the density of the particle, *C*_*D*_ is the drag coefficient, and *d*_*p*_ is the particle diameter.* Re*_*r*_ is the relative Reynolds number, which is defined as

(3) $$\begin{array}{c} Re_{r}=\frac{\rho d_{p}\left|u_{p}-u\right|}{\mu } \end{array}$$

The trajectory of the discrete phase particles can be calculated by integrating the force balance on each particle. After the iteration of each particle, the information about the position and the time is obtained. Particles with diameter ranging from 0.1 to 10.0 micrometers are simulated. We adopted the surface particles injection; i.e., a particle stream will be released from each facet of the inlet surface (the cross-sectional area). The direction of velocity of each particle is perpendicular to the facet releasing the particle, and the magnitude of velocity is the same as that of the inlet airflow. The particle tracking length scale is set as 0.00001 s, and the step length factor is 5. One-way coupling for the gas-solid flow is used [18]. Moreover, the interactions between particles are neglected because the particle flow is diluted. Our current study is a steady analysis, and the flow field is invariable. The particle is imported after the fluid solution.

Rebounds of particles do not occur since there is mucus on the airway surfaces. Hence, for all the surfaces of the airway tree model, the option of “Discrete Phase BC Type” has been set as “Trap”. It means that the trajectory calculation will be terminated when the particle is in contact with the wall, and the fate of the particle is recorded as “Trapped”. Otherwise, the particle is recorded as “Escaped” from the outlet.

### 2.4. Particle Deposition Measures

To understand the PD distribution, the deposition fraction (DF) and deposition efficiency (DE) have been defined. DF is defined as the ratio of the number of particles deposited on one region to that of particles entering the trachea, measuring the relative particle deposition number at each region. DE is defined as the ratio of the number of particles deposited on a region to that of particles entering the region, indicating the capability to capture particle of one region [21].

The accretion rate is defined as

(4) $$\begin{array}{c} R_{accretion}=\overset{N_{particle}}{\underset{p=1}{\sum }}\frac{{\dot{m}}_{p}}{A_{face}} \end{array}$$

where *N*_*particle*_ is the number of particles, *A*_*face*_ is the area of the cell face at the wall, and ${\dot{m}}_{p}$ is the mass flow rate of the particles.

In order to quantitatively analyze the deposition fraction (DF) changes in different regions of AMM model, the Deposition Enhancement Factor (DEF) has been defined as

(5) $$\begin{array}{c} DEF=\frac{{DF}_{AMM-walln}}{{DF}_{HC1-walln}} \end{array}$$

where *DF*_*AMM*−*walln*_ is the DF at* walln* in AMM model and *DF*_*HC*1−*walln*_ is the DF at* walln* in HC1 model.* n* can be T0, T1, BT, R, L, RUL, RML, RLL, LUL, and LLL, respectively. If DEF is 1, it indicates there is no change in particle deposition. If DEF is greater than 1, the deposition is enhanced; otherwise, the deposition is reduced.

### 2.5. Meshing and Grid Size Independence

The tetrahedron element and a patch independent algorithm are employed to mesh all models. The mesh quality is evaluated by the skewness; i.e., it is acceptable if the skewness is less than 0.9. In the current study, the model's skewness ranges from 0.78 to 0.87. The number of elements and nodes and the skewness of the airway models are given in Table 3.

**Figure 2:**
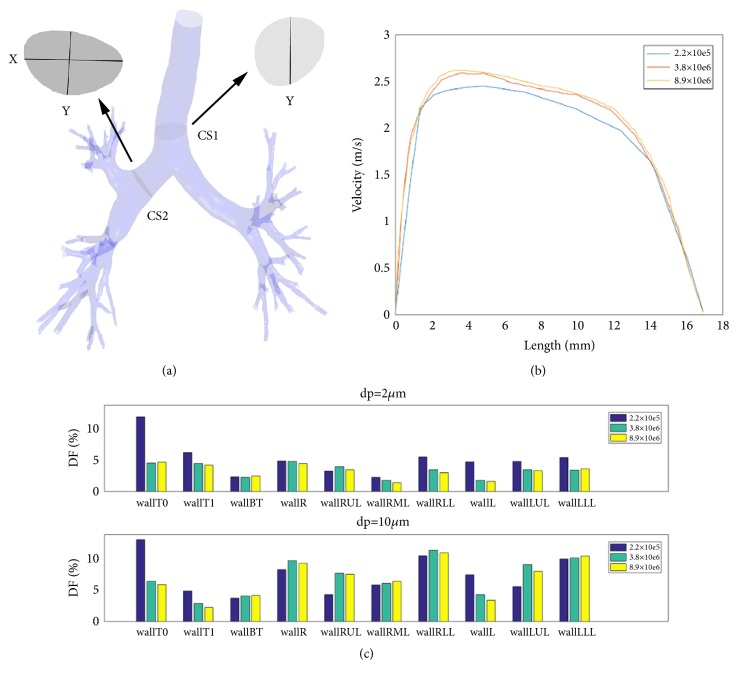
The evaluation of grids independence in HC1 model. (a) The locations of the lines and key cross-sections; (b) the velocity with different grids along line Y in CS1; (c) the deposition fraction (DF) with different grids and different particle diameters in HC1.

For studying the independence of CFD flow solution on the grid size, HC1 model is tested for three different grid sizes (with 226111, 3685470, and 8930476 elements). In Figure 2(a), two key cross-sections (CS1 and CS2) are defined within HC1 model. With all the same settings, except the grid size, the velocity profiles along one line Y at CS1 are calculated and compared. As shown in Figure 2(b), no significant difference in velocity is observed between the cases of 3685470 and 8930476 elements. The analysis of the particle deposition is shown in Figure 2(c): the value of DF is almost the same between the cases of 3685470 and 8930476 elements for different particle sizes (2 and 10.0 micrometers) and different regions (ten regions). Therefore, we adopted the scheme that contained 3685470 grids. The same scheme of controlling the grid size is adopted for all the nine models. Meanwhile, the independence of the flow velocity and the particle deposition on the grid size has been verified for each of nine models.

### 2.6. Validation of the Simulated Flow Velocity and Particle Deposition

**Figure 3:**
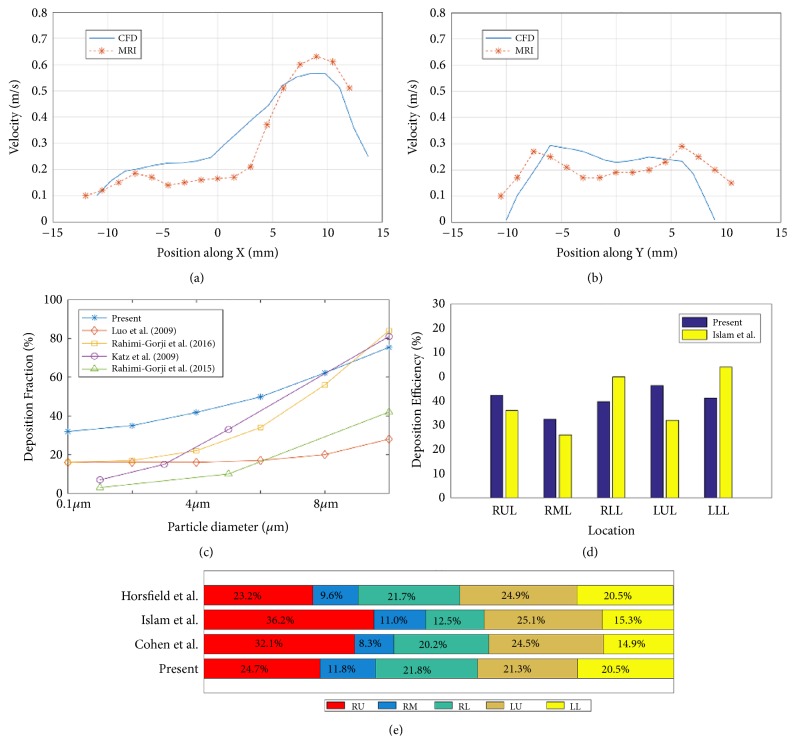
The validation of simulated flow velocity and particle deposition in HC1 model. (a) The velocity along line X in CS2 obtained by our CFD and previous MRI study; (b) the velocity along line Y in CS2 obtained by our CFD and previous MRI study; (c) the comparison of the deposition fraction (DF); (d) the comparison of the deposition efficiency; (e) the comparison of the lobar distribution of airflow.

In order to validate the airflow and particle deposition simulations of the present work, four studies have been carried out and the obtained results are compared with various published experimental data and CFD simulations. In Figures 3(a) and 3(b), the simulated flow velocity profiles at CS2 in Figure 1(a) are compared with the results achieved by the magnetic resonance gas velocimetry [10]. The inlet velocity of 0.41m/s is used in Rochefort's paper [10] and our study. The simulated velocity profiles along X and Y accord well with the measured ones. The difference of magnitude might result from geometrical variations of the two models.

Figure 3(c) shows the comparison of the overall DF between our results and those of previous studies. The flow rate is 30 liters per minute. The results of the present work have good agreement with those achieved by Rahimi-Gorji et al. [23] and Katz et al. [28]. Since the different airway tree models are used, there are some slight differences between our DF results and those obtained by Luo et al. [21] and Rahimi-Gorji et al. [22]. As shown in Figure 3(d), the higher DE at the right lower lobe and the lower DE at the right middle lobe are observed, which is also similar to the observation by Islam et al. [25]. Because of the different model structures, the DE is not the same on the specific value.  Figure 3(e) shows the lobar distribution of airflow at the flow rate of 7.5 liters per minute. The distribution is in good agreement with some previous results [25, 39, 40].

We had conducted the experiment on the independency of the number of injected particles. As an example, the results of HC1 (the DF for 2.0-micrometer particles) are described as follows. Three cases (the particle number is set as 1420, 3550, and 17750) are investigated with a steady flow rate of 26.7 L/minute. It is found that an increase of the number of particle from 1420 to 17750 does not alter the results of the DF obviously. The particle deposition is independent on the number of injected particles, which has been verified for each of nine models.

In summary, the flow velocity, DF, DE, and the lobar distribution of the airflow in the present study have been compared with the results obtained by some existing achieved works. The observed good agreement indicates that the current models and methods are sufficiently accurate to predict the PD in the airway tree models of subjects with tracheal bronchus and COPD.

## 3. Results

### 3.1. Airway Structures and Airflow Characteristics

As shown in Figure 1, the airway models of HCs are relatively smooth, indicating high ventilation efficiency. The structure of airway is individualized, i.e., the tracheal bronchus is on the wallBT in TB1 model, but on the wallR in the TB2. One stenosis occurs in wallT1 in the COPD1 model and in wallR in the COPD2 model, respectively. Boolean operations can obtain some of structures similar to those on the COPD and TB models.

**Figure 4:**
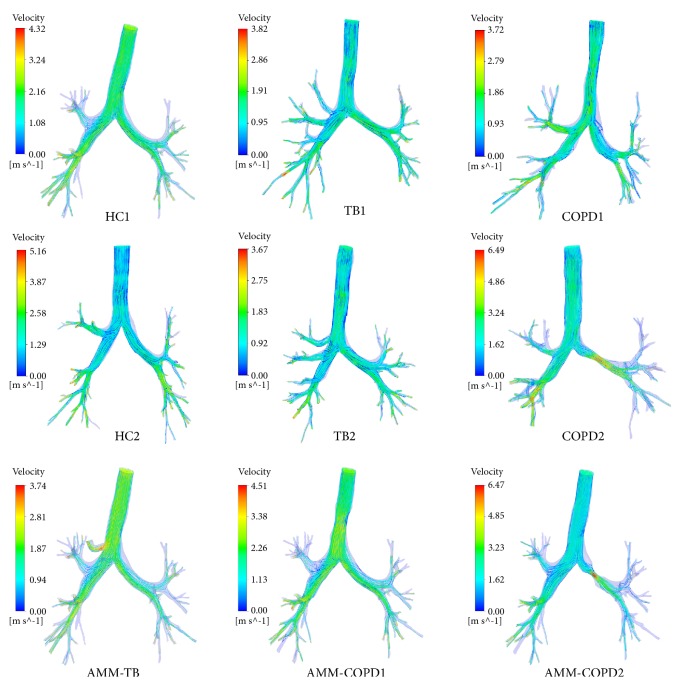
The flow velocity distributions in different airway models.

Figure 4 shows the flow velocity in nine airway tree models. In HC1 and HC2 models, there is no large eddy current, and the streamlines are smooth. For TB1 and TB2 models, the high airflow velocity is observed locally at the tracheal bronchus, but the global patterns of these measures are similar to those of HC1 and HC2. In COPD1 and COPD2 models, the large fluctuations of streamlines are available near the stenosis, and the high flow velocity is observed at the stenosis. The airflow in AMM-TB and AMM-COPD1 and AMM-COPD2 models is similar to that of TB, COPD1, and COPD2, respectively.

**Figure 5:**
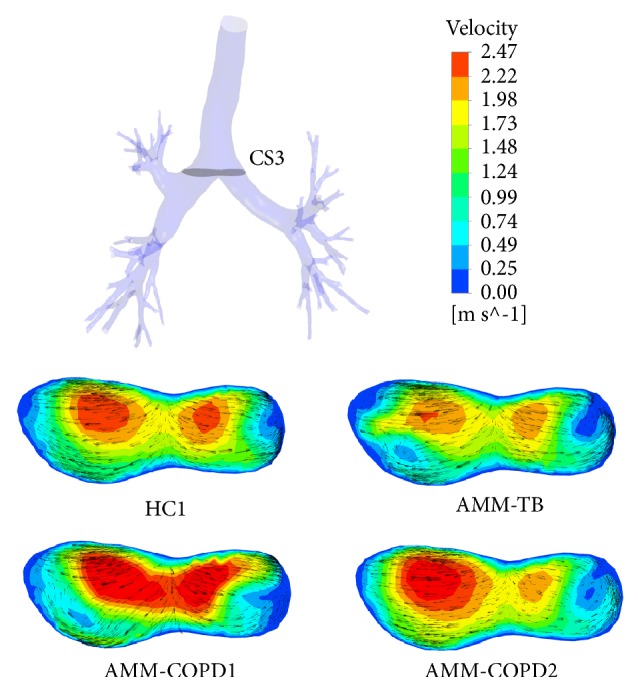
The flow velocity distributions at the key cross-section of CS3 in HC1 and three artificially modified models.

The normal and tangential components of the flow velocity at a key cross-section (CS3) are presented in Figure 5. For the normal component, the high velocity region in AMM-TB model decreases at the two main bronchi compared with HC1, which resulted from the TB. In AMM-COPD1 model, due to the stenosis, the region with high velocity at CS3 increases and the maximum velocity increases as well. In AMM-COPD2 model, the region with high velocity increases at the right side but decreases at the left because of the stenosis. The maximum velocity at the left side main bronchus decreases from 2.35 m/s to 2.12 m/s in the HC1 model. For the tangential component, the similar disruptions are also observed.

### 3.2. Particle Deposition Patterns

**Figure 6:**
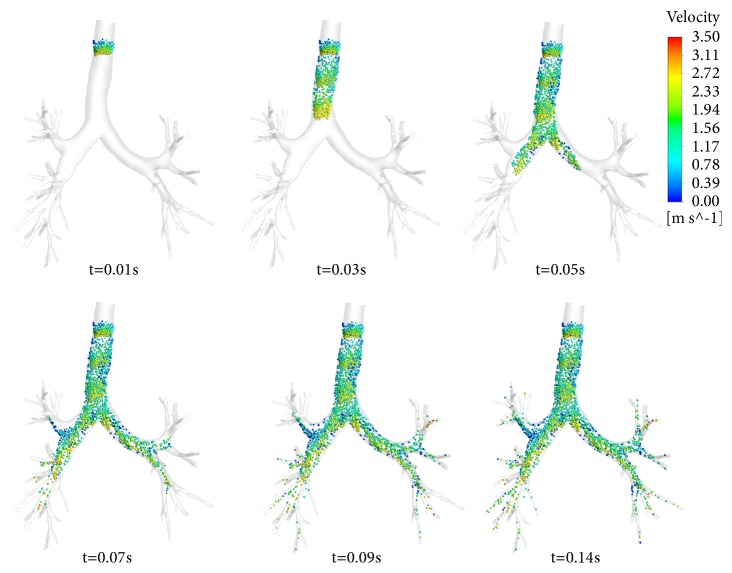
Transportation of particles at different time after injection.

Particle transport patterns are useful to illustrate the interaction of airflow structures and particle suspension.  Figure 6 depicts the particle situation at different time after injecting. The interval between particle injections is 0.02 s, and the time is at 0.01, 0.03, 0.05, 0.07, 0.09, and 0.14 s after the injection, respectively. It is found that the particle motion is influenced by the flow fluctuations, the particles velocity is enhanced, and more particles can reach the downstream airways. It is noted that more particles tend to be slowed down at the outer wall of region, caused by the combined effects of the generated centrifugal force and the secondary flow.

**Figure 7:**
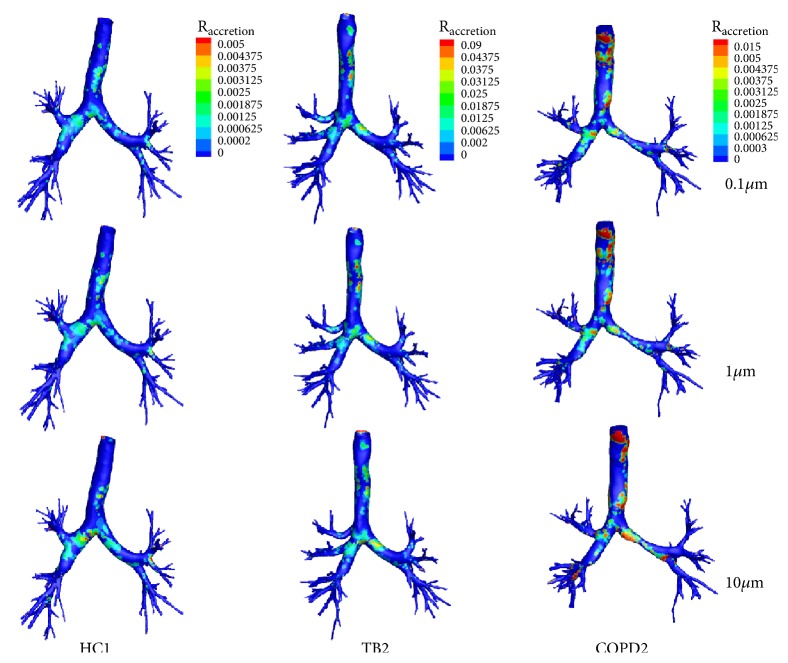
The particle deposition patterns presented by DPM accretion rate in airway tree models of HC1, TB2, and COPD2.

Figure 7 shows the PD pattern in the accretion rate with the particle size of 0.1, 1.0, and 10.0 micrometers, respectively. The four following findings have been obtained: (1) overall, the accretion rate near the bifurcation is high and most of the particles deposit on the inner bottom side. (2) The particle deposition occurs primarily on the first divider due to the direct impaction of particles. (3) The particles with the smaller size spread more uniformly and the area of particle deposition is large. With increasing particle size, the PD is concentrated on the bifurcations. (4) Comparing the results shown in Figures 4 and 7, one can find that the PD is higher at the places with large streamline fluctuations.

### 3.3. Deposition Efficiency

**Figure 8:**
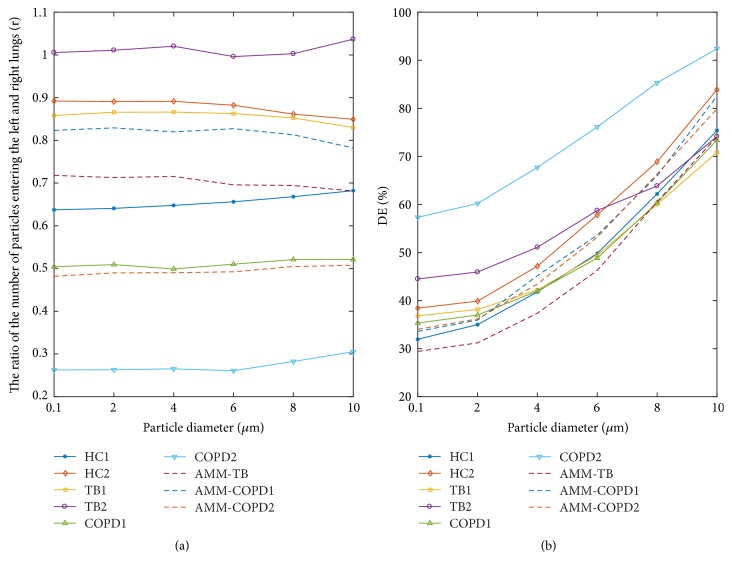
The global parameters in different airway tree models. (a) The ratio of the number of particles entering the left and right lungs; (b) the total deposition efficiency (DE) in different models.

Figure 8(a) shows the ratio of the number of particles entering the left and right lung (*r*). It can be observed that *r* is almost less than 1.0, indicating that particles tend to enter the right branch. Moreover, *r* does not change obviously with the increase of particle diameter. Among the nine models, COPD2 model has presented the smallest *r*, only 0.27 for the 2.0-micrometer particles. Meanwhile, the value of *r* in AMM-COPD2 is 0.49, less than HC1. The ratio *r* of TB1, TB2, and AMM-TB model is higher than that of HC, suggesting that the TB causes the particles to enter the left lung. HC2 model presents higher *r* than that of the HC1, which might be explained by the ratio of the angles between the trachea and the left and right main bronchus (*θ*_L_/*θ*_R_). For HC2, *θ*_L_=165.8°, *θ*_R_=157.0°, *θ*_L_/*θ*_R_=1.06; for HC1, *θ*_L_=139.4°, *θ*_R_=142.5°, *θ*_L_/*θ*_R_=0.98.

The overall DE in different models is presented in Figure 8(b). It can be seen that DE increases with the particle sizes. For example, the DE of HC1 is just 31.9% for 0.1-micrometer particles, but reaches 75.4% for 10.0-micrometer particles. In other words, the smaller particles are easier to enter the deeper airway. The COPD2 model has the highest DE, and the reason will be given in the next section. AMM-TB model has a smaller DE than that of HC1, which should be due to an additional outlet of the TB. Instead, AMM-COPD1 and AMM-COPD2 show larger DE than the HC, indicating that the stenosis increases the deposit possibility.

**Figure 9:**
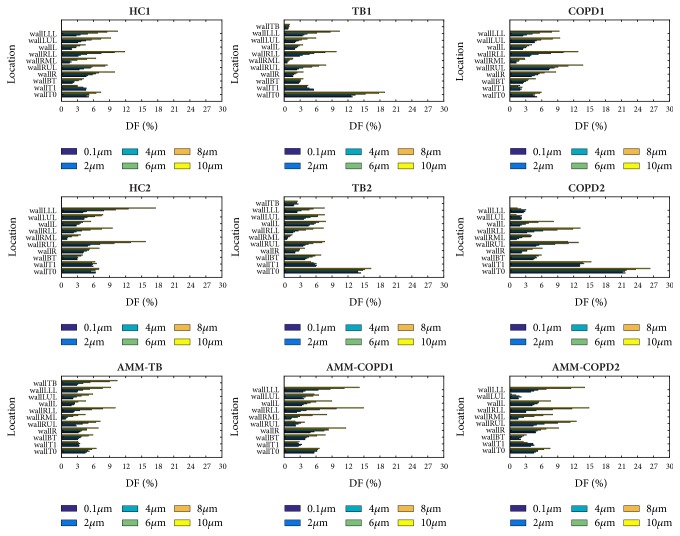
The deposition efficiency (DE) in different regions for different particle diameters.

Figure 9 illustrates the DE at different regions for the particles with various diameters. The DE of the terminal bronchi is higher than that of the central regions because the lumen area of the former one is small. DE increases with the particle size for the terminal bronchi, but remains unchanged for the central regions regardless for the particle size.

The DE of the wallTB is high for both TB models, reaching 47.4% and 63.5% for the 10.0-micrometer particles for TB1 and TB2 models, respectively. The stenosis location in the COPD model determines the effect on the DE. For instance, the DE of wallT1 in COPD1 (2.3%) is smaller than that of HC1 (4.7%) and HC2 (5.9%) because of the stenosis at wallT1 for 2.0-micrometer particles. However, the DE of wallL in COPD2 (67.0%) is larger than that of HC1 (12.9%) and HC2 (14.4%) for 10.0-micrometer particle, resulting from a stenosis at wallL. The results of AMM-TB, AMM-COPD1, and AMM-COPD2 have confirmed the above two findings.

### 3.4. Deposition Fraction

**Figure 10:**
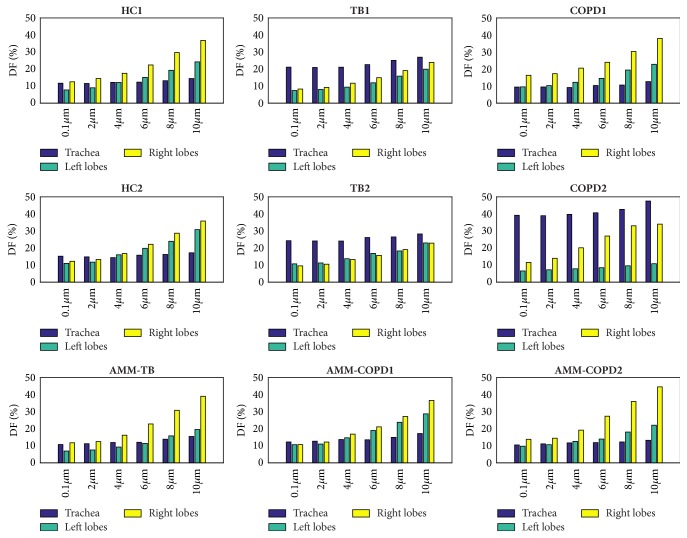
The deposition fraction (DF) on trachea, left bronchi, and right bronchi in different models.

The DF at the trachea and left and right lungs is presented in Figure 10. It is found that the DF increases with the particle diameter at right and left lungs but does not change at the trachea. The DF at the right lung is larger than that at the left lung, except for the TB2 model.

A high DF at the trachea (20.9%, 24.2%, and 38.9% for 2.0-micrometer particles) is observed in TB1, TB2, and COPD2 models compared with HC models. The irregular surface of the trachea is thought to be the reason, which is supported by the observation that the DF at the trachea of AMM-TB and AMM-COPD2 has no apparent alternation comparing with that of HC1. The DF in the left lung of COPD2 model (6.5-10.8% for 2.0-micrometer particles) is very small comparing with that of HC models. The comparison between the DF of the left lung in HC1 and AMM-COPD2 models confirms this point.

**Figure 11:**
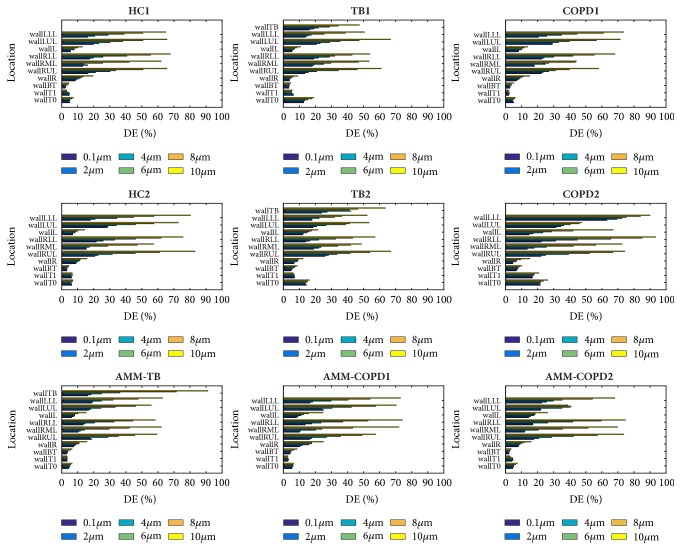
The deposition fraction (DF) in different regions for different particle diameters.

Figure 11 shows the DF of different regions across particle diameters and models. Different from DE, the DF of terminal bronchi is not higher than that of central region. The DF of terminal bronchi increases monotonically with the particle size, but the DF of central regions does not change obviously and even drops at some cases (e.g., wallT1 of HC1 model).

The DF at WallT0 of TB1, TB2, and COPD2 models is relatively high, which accords with the higher DE presented in Figure 9 and explains the higher DF at the trachea shown in Figure 11. Similar to DE, the stenosis location in COPD has different effects on DF. The DF of wallT1 in COPD1 decreases while the DF of wallBT in COPD1 increases, but the DF of wallR in COPD2 increases. The value of DEF in AMM-COPD1 and AMM-COPD2 confirmed this point. The DEF on wallT1 is 0.67, but reaches 1.67 on wallBT in AMM-COPD1 in case of 2.0-micrometer particles. In AMM-COPD2 model, the DEF in wallR reaches 2.44, indicating that the PD has been enhanced on the left main bronchus. Compared with the HC model, the DF in the right lung of AMM-TB becomes smaller, the DF in WallR, WallRUL, and WallLUL decreases from 9.9%, 8.5%, and 11.8% to 6.8%, 7.2%, and 10.0% for the 10.0-micrometer particles, respectively. This decrease can be explained by the existence of the TB.

## 4. Discussions

This is the first work of studying PD in the realistic airway tree models of subjects with TB and COPD. Meanwhile three AMMs have been used to only evaluate the effect of TB and stenosis. The most important finding is that the PD in airway with TB and COPD has been disrupted by the changes of the airway geometry leading to alteration of the airflow.

For both TB models, the simulation showed that the DE of the TB is relatively high. It suggests that the particles of pollutants are easier to deposit at the TB. Combined with the high airflow velocity, wall pressure, and wall shear stress presented locally at the tracheal bronchus [32], the high DE might explain why inflammation is more likely to occur at the TB than in other regions [4]. Nevertheless, the high DE also has a good effect on the treatment of the TB lesions using aerosol medicine [30, 41].

For COPD models, the location of the stenosis determines the effects on DE and DF. If the stenosis appears in the left main bronchus, DE will increase at this region, which accords with previous studies [15]. It also supports the hypothesis that long-term PM exposures increase symptoms of obstructive airway disease [1]. The DF in the left upper and left lower lobes will decrease if the stenosis occurs on the left main bronchus [6]. The personalized deposition patterns presented in our study emphasize the need for optimizing inhalation therapies using CFD method and CT images [30].

Compared with HC, a large number of particles deposit on the tracheae in TB1, TB2, and COPD2 models. Irregular surface of the trachea is prone to PD [22]. It is suggested that new nebulizers are required if the lesion is at the terminal bronchi.

There are at least three advanced methodological features in our current study. First, the airway tree model is extracted from the CT images, which makes the CFD simulation provide with rich PD information at a personalized level with multiple variables. Second, the LRN k–*ω* turbulence model is used, which has been proved to be very efficient by previous some studies [18, 21, 36]. Third, by comparing the PD on airway model of one specific individual with that on its corresponding AMM model, the impact of the airway geometry differences between different individuals on the PD can be excluded, and the impact of virtual lesion can be emphasized separately. This virtual lesion method was used to produce the three asthmatic models [42], and virtual interventions were employed to design personalized surgical planning [43]. These features enable CFD to open new pathways of further optimization of the drug delivery and respiratory devices [30].

There are some limitations in the current study. First, the sample size is small, only six participants are involved. The findings might not exactly represent the characteristics of the whole group. The group studies will be conducted in the future, just like done by De Backer et al. [13], Bos et al. [30], and Katz et al. [28]. Second, the CFD simulated results have not been validated by the direct experiments though the validation has been done [10]. Third, one constant flow rate is adopted in current study. Multiple flow rates or the subject-specific flow rates are recommended [44]. Finally, the steady inlet velocity and the rigid airway wall are used in our study. The fluid-solid interaction and transient airflow model should be adopted [27, 34, 45].

## 5. Conclusions

The particle deposition in subjects with TB and COPD is disrupted by the pathologically geometric alternations of airway. Combined with artificially modified models, CFD simulation using realistic airway model can demonstrate these disruptions. The method and findings might help understand the etiology of pulmonary diseases from the viewpoint of particle deposition and improve treatment efficacy of inhaled medicines.

## Figures and Tables

**Table 1:** Subject information, CT image scanning, and reconstruction parameters.

ID	Gender	Age	Tube current (mAs)	FOV (mm)	Pixel size (mm)	Number of slices	CT scanner
TB1	Male	41	212.89	388.00	0.758	360	Philips Ingenuity Core 128
TB2	Male	27	114.93	377.00	0.736	335	Philips Ingenuity Core 128
COPD1	Male	61	91.50	355.33	0.694	401	TOSHIBA Aquilion ONE
COPD2	Male	53	134.92	380.00	0.742	300	Philips Ingenuity Core 128
HC1	Female	57	139.93	350.00	0.684	366	Philips Ingenuity Core 128
HC2	Male	66	118.92	350.00	0.684	375	Philips Ingenuity Core 128

**Table 2:** The morphometric parameters in TB COPD and HC model.

	HC1	HC2	TB1	TB2	COPD1	COPD2
Volume(mm^3^)	46453.2	51984.9	67740.2	75282.2	83446.5	42577.8
Surface area(mm^2^)	20377.2	22424.8	28162.0	29790.3	32901.7	20221.9
Inlet area (mm^2^)	185.4	305.9	317.0	297.9	319.4	236.3

**Table 3:** The statistic parameters of mesh of the airway models.

ID	Number of elements	Number of nodes	Skewness
TB1	4099891	816514	0.786
TB2	3768926	753281	0.793
COPD1	4001057	798293	0.813
COPD2	4122246	822222	0.867
HC1	3685470	737137	0.845
HC2	3752108	753347	0.809
AMM-TB	3710702	742154	0.828
AMM-COPD1	3748164	749429	0.853
AMM-COPD2	3725895	745311	0.880

## Data Availability

The CT images and related CFD models used to support the findings of this study are available from the corresponding author upon request.

## Nomenclature

*θ*_L_: The ratio of the angles between the trachea and the left main bronchus
*θ*_R_: The ratio of the angles between the trachea and the right main bronchus
${\dot{m}}_{p}$: The mass flow rate of particles
*A*_*face*_: The area of the cell face at the wall
*DF*_*AMM*−*walln*_: The DF at* walln* on AMM model
*DF*_*HC*1−*walln*_: The DF at* walln* on HC1 model
*F*_*D*_: The drag force
*F*_*x*_: An additional acceleration term
*N*_*particle*_: The number of particles
*R*_*accretion*_: The accretion rate
*u*_*p*_: The particle velocity
*C*_*D*_: The drag coefficient
DE: Deposition efficiency
DEF: The deposition enhancement factor
DF: Deposition fraction
*d*_*p*_: The particle diameter
*r*: The ratio of the number of particles entering the left and right lung
*Re*_*r*_: The relative Reynolds number
*u*: The fluid velocity
*μ*: The dynamic viscosity of the fluid
*ρ*: The fluid density
*ρ*_*p*_: The density of the particle.

## Supplementary Materials

Supplementary Materials This video shows the transportation and deposition of particles with a diameter of 10.0 microns in the realistic airway tree model of one subject with chronic obstructive pulmonary disease (COPD). The model is extracted from the COPD subject's CT images. The legend indicates the flow velocity of particles. The initial velocity of particles is set as 1.96 m/s according to the inlet flow rate (26.7 liters per minute) and the inlet area. The interval between particle injections is 0.02 seconds. In the COPD model, there is one stenosis at the left main bronchus. From this video, the alternations of the transportation and deposition of particles resulted from this stenosis can be presented at the upstream and downstream of the stenosis
